# Famitinib versus placebo in the treatment of refractory metastatic colorectal cancer: a multicenter, randomized, double-blinded, placebo-controlled, phase II clinical trial

**DOI:** 10.1186/s40880-017-0263-y

**Published:** 2017-12-22

**Authors:** Rui-Hua Xu, Lin Shen, Ke-Ming Wang, Gang Wu, Chun-Mei Shi, Ke-Feng Ding, Li-Zhu Lin, Jin-Wan Wang, Jian-Ping Xiong, Chang-Ping Wu, Jin Li, Yun-Peng Liu, Dong Wang, Yi Ba, Jue-Ping Feng, Yu-Xian Bai, Jing-Wang Bi, Li-Wen Ma, Jian Lei, Qing Yang, Hao Yu

**Affiliations:** 1 Department of Medical Oncology, Sun Yat-sen University Cancer Center, State Key Laboratory of Oncology in South China, Collaborative Innovation Center for Cancer Medicine, Guangzhou, 510060 Guangdong P. R. China; 2 Key Laboratory of Carcinogenesis and Translational Research (Ministry of Education), Department of Gastrointestinal Oncology, Peking University Cancer Hospital & Institute, No 52, Fucheng Road, Haidian District, Beijing, 100142 P. R. China; 3 Department of Medical Oncology, Second Affiliated Hospital of Nanjing Medical University, Nanjing, 210011 Jiangsu P. R. China; 4 Cancer Center of Union Hospital, Tongji Medical College, Huazhong University of Science and Technology, Wuhan, 430030 Hubei P. R. China; 5 Department of Medical Oncology, Fujian Medical University Union Hospital, Fuzhou, 350001 Fujian P. R. China; 6 Department of Surgical Oncology, Second Hospital Affiliated to Zhejiang University School of Medicine, Hangzhou, 310009 Zhejiang P. R. China; 7 Department of Oncology, First Affiliated Hospital of Guangzhou Medical University of Chinese Medicine, Guangzhou, 510405 Guangdong P. R. China; 8 Department of Medical Oncology, Chinese Academy of Medical Sciences Cancer Hospital, Beijing, 100021 P. R. China; 9 Department of Medical Oncology, First Affiliated Hospital of Nanchang University, Nanchang, 330006 Jiangxi P. R. China; 10 Department of Medical Oncology, First People’s Hospital of Changzhou, Changzhou, 213003 Jiangsu P. R. China; 11 Department of Medical Oncology, Fudan University Cancer Hospital, Shanghai, 200032 P. R. China; 12 Department of Medical Oncology, First Hospital of China Medical University, Shenyang, 110001 Liaoning P. R. China; 13 Cancer Center, Daping Hospital and Institute of Surgery Research, Third Military Medical University, Chongqing, 400042 P. R. China; 14 Department of Gastrointestinal Medical Oncology, Tianjin Medical University Cancer Institute and Hospital, Tianjin, 300060 P. R. China; 15 Department of Oncology, PuAi Hospital of Tongji Medical College, Huazhong University of Science and Technology, Wuhan, 430032 Hubei P. R. China; 16 Department of Medical Oncology, Harbin Medical University Cancer Hospital, Harbin, 150081 Heilongjiang P. R. China; 17 Department of Oncology, Jinan Military General Hospital, Jinan, 250000 Shandong P. R. China; 18 Department of Tumor Chemotherapy and Radiology, Peking University Third Hospital, Beijing, 100191 P. R. China; 19 Department of Gastrointestinal Surgery, First Affiliated Hospital of Guangzhou Medical University, Guangzhou, 510120 Guangdong P. R. China; 20 Department of Clinical Medicine, Jiangsu Hengrui Medicine Co., Ltd, Lianyungang, 222047 Jiangsu P. R. China; 21 Department of Epidemic and Health Statistics, Nanjing Medical University, Nanjing, 211166 Jiangsu P. R. China

**Keywords:** Colorectal cancer, Famitinib, Efficacy, Safety

## Abstract

**Background:**

Metastatic colorectal cancer (mCRC) patients with progressive disease after all available standard therapies need new medication for further treatment. Famitinib is a small-molecule multikinase inhibitor, with promising anticancer activities. This multicenter, randomized, double-blinded, placebo-controlled, phase II clinical trial was designed to evaluate the safety and efficacy of famitinib in mCRC.

**Methods:**

Famitinib or placebo was administered orally once daily. The primary endpoint was progression-free survival (PFS). Secondary endpoints included objective response rate (ORR), disease control rate (DCR), overall survival (OS), quality-of-life (QoL), and safety.

**Results:**

Between July 18, 2012 and Jan 22, 2014, a total of 167 patients were screened, and 154 patients were randomized in a 2:1 ratio to receive either famitinib (*n* = 99) or placebo (*n* = 55). The median PFS was 2.8 and 1.5 months in the famitinib and placebo groups (hazard ratio = 0.60, 95% confidence interval = 0.41–0.86, *P* = 0.004). The DCR was 59.8% and 31.4% (*P* = 0.002) and the ORR was 2.2% and 0.0% (*P* = 0.540) in the famitinib and placebo groups, respectively. The most frequent grade 3–4 adverse events were hypertension (11.1%), hand-foot syndrome (10.1%), thrombocytopenia (10.1%), and neutropenia (9.1%). Serious adverse events occurred in 11 (11.1%) patients in the famitinib group and 5 (9.1%) in the placebo group (*P* = 0.788). The median OS of the famitinib and placebo groups was 7.4 and 7.2 months (*P* = 0.657).

**Conclusion:**

Famitinib prolonged PFS in refractory mCRC patients with acceptable tolerability.

*Trial registration* This study was registered on ClinicalTrials.gov (NCT01762293) and was orally presented in the 2015 ASCO-Gastrointestinal Symposium

## Background

Colorectal cancer (CRC) is a common malignancy and the second leading cause of cancer-related deaths worldwide [1]. At least half of patients will eventually develop metastases [2, 3]. Moreover, the incidence and mortality of CRC have been rising quickly in recent years in China [4]. Combination chemotherapy, consisting of 5-fluorouracil (5-FU) or oral 5-FU analogues, irinotecan and oxaliplatin, with or without anti-epidermal growth factor receptor (anti-EGFR) and anti-angiogenesis monoclonal antibody, are adopted as the standard first- or second-line therapy for CRC [5–9]. However, there are no other drugs except for regorafenib being used to treat metastatic CRC (mCRC) after standard chemotherapy failure. Regorafenib has been approved to treat mCRC based on the results of two phase III studies CORRECT [10] and CONCUR [11]. Another clinical study involving fruquintinib also demonstrated promising anticancer effect on mCRC [12].

Famitinib (famitinib l-malate) is a novel and potent receptor tyrosine kinase inhibitor (rTKI) [13]. The targets of famitinib include tyrosine kinase receptor c-kit, vascular endothelial growth factor receptor-2 and -3 (VEGFR-2 and -3), platelet-derived growth factor receptor (PDGFR), FMS-like tyrosine kinase-3 receptor (FLT3), and tyrosine-protein kinase receptor Ret [13, 14]. A phase I study showed that famitinib was generally well-tolerated and has a wide spectrum of antitumor activities [14]. Based on the results of famitinib from pre-clinical and phase I studies and due to the high unmet needs of Chinese mCRC patients, we initiated a multicenter, randomized, double-blinded, placebo-controlled, phase II study to evaluate the efficacy and safety of famitinib in Chinese patients with mCRC who failed standard therapies.

## Patients and methods

### Patients and study design

This clinical trial involves 19 hospitals/institutions in China. The clinical trial protocol was approved by the institutional review board of each center.

The inclusion criteria are as follows: (1) patients have pathologically confirmed advanced colorectal adenocarcinoma (excluding all other histological types) and have previously received at least two lines of standard chemotherapy (must include 5-FU, irinotecan, and oxaliplatin) and failed treatment (treatment failure is defined as intolerable adverse events [AEs] or disease progression during treatment or within 3 months after the last treatment); (2) according to the response evaluation criteria in solid tumors (RECIST) version 1.1 criteria [15], patients must have at least one target lesion with measurable diameter (long diameter of tumor lesion ≥ 10 mm and short diameter of lymph node lesion ≥ 15 mm on computed tomography [CT], with scan slice thickness no more than 5 mm; without local treatment); (3) age of 18–70 years; (4) Eastern Cooperative Oncology Group (ECOG) performance status score of 0 or 1; and (5) life expectancy ≥ 3 months.

The exclusion criteria are as follows: (1) with a history or presence of other malignancies, excluding cured skin basal cell carcinoma and carcinoma in situ of the cervix; (2) with previous treatment with VEGFR TKIs (e.g., sorafenib, sunitinib, and regorafenib); (3) with multiple factors influencing oral administration (e.g., inability to swallow, chronic diarrhea, and intestinal obstruction); (4) with definite gastrointestinal bleeding tendency evidenced by local active ulcer lesions and stool occult blood (++), history of melena and hematemesis in the past 2 months, and potential of major gastrointestinal bleeding considered by the investigator; (5) with evidence of central nervous system (CNS) metastasis at baseline or a history of CNS metastasis (for patients with clinically suspected CNS metastasis, CT or magnetic resonance imaging [MRI] scan must be performed within 14 days prior to randomization to exclude CNS metastasis); (6) excessive tumor burden of vital organs (e.g., liver tumor burden > 50%) demonstrated with imaging; or (7) abnormal function of vital organs. Each subject provided written informed consent before enrollment.

### Randomization and blinding

Patients were randomly assigned to the famitinib or placebo group in a 2:1 ratio. A centralized randomization system, supplied by the Department of Epidemiology and Health Statistics at Nanjing Medical University, was used. Randomization of subjects was on the basis of pre-allocated block sizes (block size six) and was stratified by previous treatment (no more than or more than three-line therapy) and baseline lactate dehydrogenase (LDH) level (≤ 1.5 or > 1.5 times of the upper limit of normal).

The unblinding of treatment for individual patients was allowed for emergency situations only.

### Treatment and follow-up

Patients were treated with 25 mg oral famitinib or matching placebo tablet once daily until progressive disease (PD), death, unacceptable AEs, withdrawal of consent by the patient or a decision by the physician that discontinuation would be in the patient’s best interest. Patients were followed-up every 2 weeks for the first 6 weeks and every 3 weeks thereafter while receiving treatment, and every 6 weeks after cessation of treatment until death or the last follow up of August 21, 2014. Predefined dose modifications were permitted to manage clinically significant treatment-related AEs. This study was conducted in compliance with ethical principles originated or derived from the Declaration of Helsinki (October 1996).

### Assessments

Tumor response was assessed radiologically every 6 weeks, using the RECIST version 1.1 criteria [15].

The primary endpoint was progression-free survival (PFS), defined as the duration from treatment initiation to first radiological observation of PD or death from any cause. Secondary endpoints included overall survival (OS), defined as the duration from treatment initiation to death from any cause; objective tumor response rate (ORR), defined as the proportion of patients with complete or partial response; disease control rate (DCR), defined as the proportion of patients with a best response of complete or partial response or stable disease (defined as disease stabled for more than 6 weeks after randomization); and quality of life (QoL) evaluated by questionnaire and safety assessments.

Patients’ health-related QoL and health utility values were measured before enrollment and at the end of each 6-week treatment, according to the European Organization for Research and Treatment of Cancer (EORTC) general health status and quality of life questionnaire QLQ-C30 [16]. AEs were graded with the National Cancer Institute Common Terminology Criteria for Adverse Events (Version 4.0) [17].

### Statistical analysis

Based on the investigators’ opinions, the assumed median PFS of patients in the famitinib and placebo groups would be 3.2 and 2.0 months, respectively. Considering that this was a phase II trial, setting one-sided significant level to 0.05, with a famitinib-to-placebo allocation ratio of 2:1, 144 subjects in total could achieve 80% power.

All statistical analyses were performed using SAS version 9.2 (SAS Institute, Cary, NC, USA). Median OS and PFS with 95% confidence interval (CI) for each group were estimated using the Kaplan–Meier method. Patients without disease progression or death by the last follow-up would be censored in PFS or OS curves. OS and PFS were compared between the famitinib and placebo groups using the log-rank test. Stratified log-rank tests by previous treatment and LDH level were also performed. If the proportionality assumption holds true, Cox proportional hazard model was used to estimate the hazard ratio (HR) and 95% CI with treatment as a fixed factor. Cox proportional hazard model adjusted for stratification factors including age, gender, LDH level, number of metastatic organs and treatment line was also performed. ORR and DCR were compared between treatment groups using the Cochran–Mantel–Haenszel test, adjusted for stratification factors. Baseline characteristics and AEs were compared with the use of analysis of variance or Chi square test as appropriate.

## Results

### Patient characteristics

**Fig. 1:**
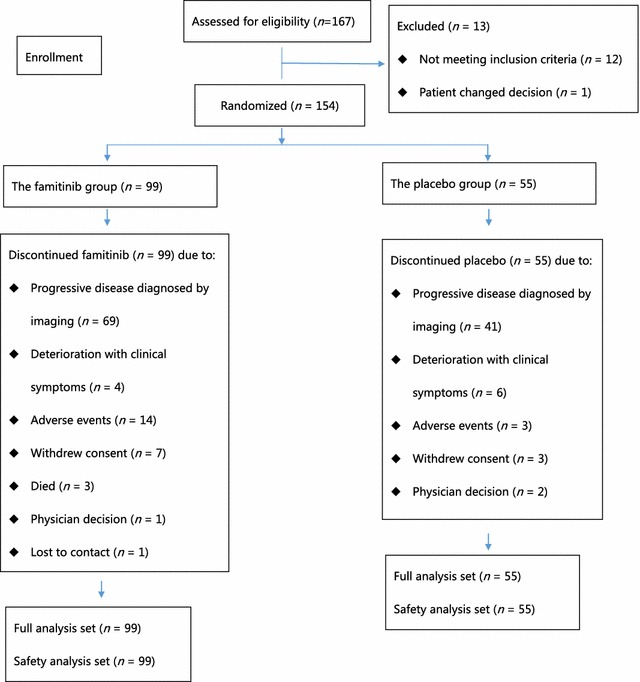
The flow diagram of patient enrollment for the phase II clinical trial of famitinib versus placebo in the treatment of refractory metastatic colorectal cancer (mCRC)

Between July 18, 2012 and Jan 22, 2014, 154 patients were enrolled and randomized to receive famitinib (*n* = 99) or placebo (*n* = 55), and the flow diagram is shown in Fig. 1. The baseline characteristics were balanced between the two groups (Table 1). Overall, 71 (46.1%) of the 154 patients had previously received monoclonal antibody treatment, and 94 (61.0%) had received more than three lines of treatment for mCRC.

**Table 1:** Baseline characteristics of patients with metastatic colorectal cancer (mCRC) who were treated with famitinib or placebo in the full analysis set

Characteristic	Whole cohort	Famitinib group	Placebo group	*P* value
Total (cases)	154	99	55	
Age (years)				0.352
Mean ± SD	54.1 ± 9.5	54.7 ± 9.9	53.2 ± 8.8	
Median (range)	55 (24–71)	55 (24–70)	54 (32–71)	
Age group [cases (%)]				0.437
> 60 years	106 (68.8)	66 (66.7)	40 (72.7)	
≤ 60 years	48 (31.2)	33 (33.3)	15 (27.3)	
Gender [cases (%)]				0.679
Male	89 (57.8)	56 (56.6)	33 (60.0)	
Female	65 (42.2)	43 (43.4)	22 (40.0)	
ECOG performance status [cases (%)]				0.776
0	27 (17.5)	18 (18.2)	9 (16.4)	
1	127 (82.5)	81 (81.8)	46 (83.6)	
LDH level [cases (%)]				0.663
≤ 1.5 × ULN	126 (81.8)	80 (80.8)	46 (83.6)	
> 1.5 × ULN	28 (18.2)	19 (19.2)	9 (16.4)	
Number of metastatic organs [cases (%)]				0.499
≤ 2	84 (54.5)	56 (56.6)	28 (50.9)	
> 2	70 (45.5)	43 (43.4)	27 (49.1)	
Primary site of disease [cases (%)]				0.485
Rectum	73 (47.4)	49 (49.5)	24 (43.6)	
Colon	81 (52.6)	50 (50.5)	31 (56.4)	
History of primary tumor resection [cases (%)]				0.559
No	14 (9.1)	10 (10.1)	4 (7.3)	
Yes	140 (90.9)	89 (89.9)	51 (92.7)	
History of anti-tumor monoclonal antibody therapy [cases (%)]				0.579
No	83 (53.9)	55 (55.6)	28 (50.9)	
Yes	71 (46.1)	44 (44.4)	27 (49.1)	
Famitinib treatment as [cases (%)]				0.622
Third-line therapy	60 (39.0)	40 (40.4)	20 (36.4)	
> Third-line therapy	94 (61.0)	59 (59.6)	35 (63.6)	
White blood cell count (10^9^/L)				0.646
Mean ± SD	6.6 ± 2.5	6.7 ± 2.4	6.5 ± 2.7	
Median (range)	6.1 (3.0–17.0)	6.2 (3.0–15.0)	5.8 (3.0–17.0)	
Alkaline phosphatase (U/L)				0.887
Mean ± SD	127.3 ± 82.8	126.6 ± 82.6	128.6 ± 84.0	
Median (range)	104.0 (26.0–580.0)	105.0 (26.0–580.0)	103.0 (46.0–458.0)	

*SD* standard deviation, *ECOG* Eastern Cooperative Oncology Group, *LDH* lactate dehydrogenase, *ULN* upper limit of normal

### Treatment situation

The mean duration of treatment was 86.8 ± 78.0 days (median 63.0 days; interquartile range [IQR] 40.0–105.0 days) for the famitinib group and 58.2 ± 47.1 days (median 42.0 days; IQR 40.0–82.0 days) for the placebo group. The mean duration of follow-up from the completion of study treatment to last follow-up was 6.2 ± 4.4 months (median 5.1 months; IQR 2.9–9.2 months) for the famitinib group and 7.3 ± 5.5 months (median 5.4 months; IQR 2.9–10.0 months) for the placebo group.

The mean daily dose of famitinib was 23.1 ± 3.2 mg (median 25.0 mg; IQR 20.9–25.0 mg). The mean daily dose of placebo was 24.8 ± 1.4 mg (median 25.0 mg; IQR 25.0–25.0 mg).

As shown in Table 2, dose interruption occurred in 49 (49.5%) patients and dose reduction occurred in 24 (24.2%) patients in the famitinib group; however, in the placebo group, dose interruption occurred in 13 (23.6%) patients and dose reduction occurred in 2 (3.6%) patients. AEs were the most common reasons for dose modification.

**Table 2:** Treatment modification based on patients’ tolerance during the study in the safety analysis set

Treatment modification	Famitinib group [cases (%)]	Placebo group [cases (%)]
Treatment interruption	49 (49.5)	13 (23.6)
Once	26 (26.3)	12 (21.8)
Twice	8 (8.1)	1 (1.8)
More than twice	15 (15.2)	0
Dose reduction	24 (24.2)	2 (3.6)
One reduction	19 (19.2)	1 (1.8)
Two reductions	5 (5.1)	1 (1.8)

### Clinical outcome

At last, 92 patients in the famitinib group and 51 in the placebo group had clinical response evaluation. Complete response was not achieved in any group. Two patients in the famitinib group had partial response (ORR = 2.2%), compared to none in the placebo group (*P* = 0.540). DCR was 59.8% (55/92) in the famitinib group and 31.4% (16/51) in the placebo group (*P* = 0.002).

**Fig. 2:**
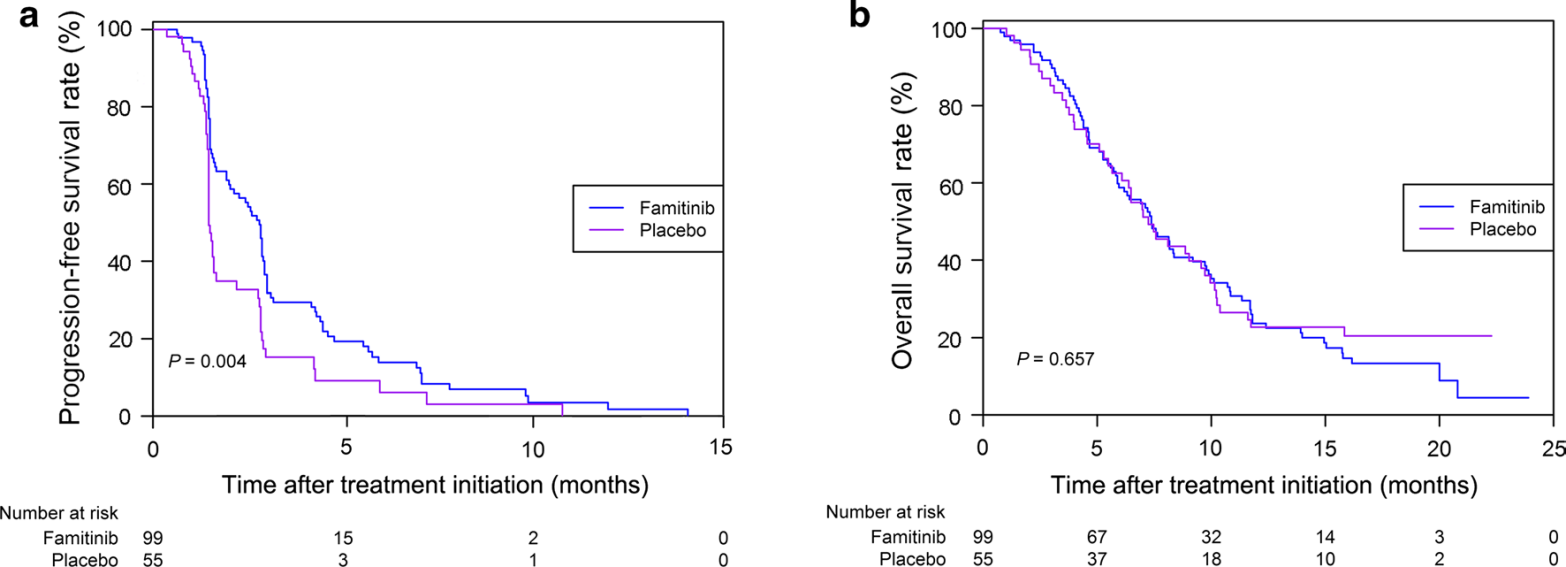
Kaplan–Meier estimates of progression-free survival (PFS) and overall survival (OS) probability of mCRC patients treated with famitinib and placebo. **a** the median PFS in the famitinib group was significantly longer than that in the placebo group (*P* = 0.004); **b** there is no significant difference in the median OS between the two groups

**Fig. 3:**
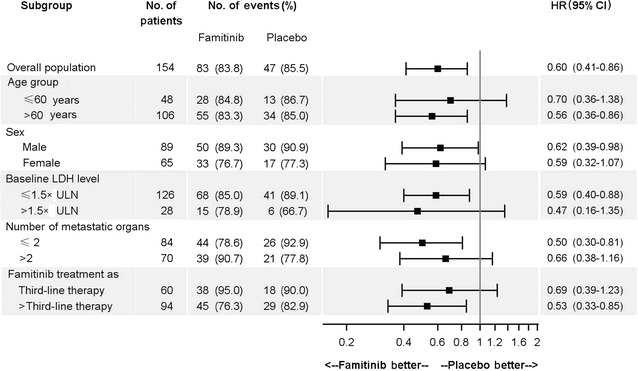
Factors associated with PFS of mCRC patients as identified by stratified analysis. *LDH* lactate dehydrogenase, *ULN* upper limit of normal, *HR* hazard ratio, *95% CI* 95% confidence interval

Up to the last follow-up, disease progression and death occurred in 83 (83.8%) and 82 (82.8%) patients in the famitinib group and in 47 (85.5%) and 42 (76.4%) patients in the placebo group. The median PFS was 2.8 and 1.5 months in the famitinib and placebo groups (HR = 0.60, 95% CI = 0.41–0.86, *P* = 0.004, Fig. 2a), and the median OS was 7.4 and 7.2 months in the famitinib and placebo groups (*P* = 0.657, Fig. 2b). Stratified analysis including age, gender, LDH level, number of metastatic organs, and treatment line demonstrated that the famitinib group had longer PFS than the placebo group (Fig. 3).

Following famitinib or placebo treatment, 31 (31.3%) and 23 (41.8%) patients in the famitinib and placebo groups received further anti-cancer therapies. As shown in Table 3, 21 (21.2%) patients in the famitinib group and 6 (10.9%) patients in the placebo group received chemotherapy, including S-1, raltitrexed, or other regimens.

**Table 3:** The further anti-tumor therapy following the completion of famitinib or placebo treatment in the full analysis set

Further therapy	Famitinib group [cases (%)] (*n* = 99)	Placebo group [cases (%)] (*n* = 55)
Chemotherapy	21 (21.2)	6 (10.9)
Traditional Chinese medicine	4 (4.0)	4 (7.3)
McAb	0 (0.0)	7 (12.7)
Chemotherapy plus McAb	2 (2.0)	3 (5.5)
Cellular immunotherapy	0 (0.0)	2 (3.6)
Cellular immunotherapy plus chemotherapy	1 (1.0)	0 (0.0)
Targeted therapy	0 (0.0)	1 (1.8)
Radiotherapy	3 (3.0)	2 (3.6)
Radiochemotherapy	1 (1.0)	1 (1.8)
Surgery	2 (2.0)	1 (1.8)
Interventional therapy	2 (2.0)	0 (0.0)
Unspecified	6 (6.1)	2 (3.6)

*McAb* monoclonal antibody

**Fig. 4:**
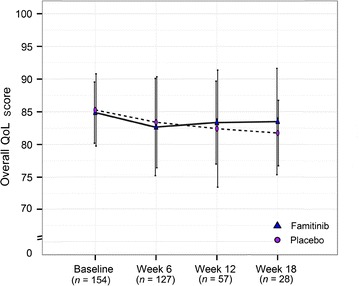
Mean overall quality of life (QoL) score of mCRC patients over early study visits at baseline and during famitinib or placebo treatment. All data points are presented as mean ± standard deviation

Using the EORTC questionnaire QLQ-C30, the mean QoL scores at baseline were 84.9 ± 4.7 in the famitinib group and 85.3 ± 5.5 in the placebo group; the mean scores at the end of study treatment were 83.5 ± 8.1 in the famitinib group and 81.7 ± 5.0 in the placebo group (Fig. 4), indicating a mild decrease in QoL in both groups. Patients’ QoL and health status deteriorated to a similar extent in both groups (*P* > 0.100 for all visit-specific comparisons; *P* = 0.534 for overall comparison).

### Safety

Table 4 shows treatment-related AEs that occurred in the two groups during the study. The most frequent AEs in the famitinib group were hematologic toxicity, proteinuria, hypertension, hand-foot syndrome, diarrhea, fatigue, hand-foot skin reaction, and liver dysfunction. In the placebo group, the most frequent AEs were liver dysfunction, fatigue, and proteinuria.

**Table 4:** Treatment-related adverse events occurring in mCRC patients during the study in the safety analysis set

Adverse event	Famitinib group [cases (%)] (*n* = 99)		Placebo group [cases (%)] (*n* = 55)	
	Any grade	Grade 3–4	Any grade	Grade 3–4
Any event^a^	92 (92.9)	51 (51.5)	42 (76.4)	20 (36.4)
Clinical adverse event				
Hypertension	38 (38.4)	11 (11.1)	4 (7.3)	1 (1.8)
Hand-foot syndrome	27 (27.3)	10 (10.1)	1 (1.8)	0 (0.0)
Diarrhea	15 (15.2)	1 (1.0)	3 (5.5)	0 (0.0)
Fatigue	14 (14.1)	3 (3.0)	9 (16.4)	2 (3.6)
Rash	8 (8.1)	2 (2.0)	0 (0.0)	0 (0.0)
Hypothyroidism	7 (7.1)	0 (0.0)	1 (1.8)	0 (0.0)
Oral mucositis	7 (7.1)	0 (0.0)	0 (0.0)	0 (0.0)
Nausea	6 (6.1)	0 (0.0)	4 (7.3)	1 (1.8)
Decrease appetite	6 (6.1)	0 (0.0)	5 (9.1)	0 (0.0)
Headache	6 (6.1)	1 (1.0)	2 (3.6)	1 (1.8)
Vomit	5 (5.1)	1 (1.0)	2 (3.6)	1 (1.8)
Dizziness	5 (5.1)	0 (0.0)	2 (3.6)	0 (0.0)
Backache	3 (3.0)	0 (0.0)	3 (5.5)	0 (0.0)
Abdominal distension	2 (2.0)	0 (0.0)	3 (5.5)	0 (0.0)
Cough	2 (2.0)	0 (0.0)	5 (9.1)	0 (0.0)
Laboratory abnormalities				
Proteinuria	42 (42.4)	6 (6.1)	9 (16.4)	0 (0.0)
Neutropenia	41 (41.4)	9 (9.1)	1 (1.8)	1 (1.8)
Leukopenia	36 (36.4)	3 (3.0)	1 (1.8)	0 (0.0)
Thrombocytopenia	31 (31.3)	10 (10.1)	1 (1.8)	1 (1.8)
Increased γ-GT	20 (20.2)	7 (7.1)	11 (20.0)	7 (12.7)
Increased ALT	17 (17.2)	3 (3.0)	8 (14.5)	1 (1.8)
Increased AST	16 (16.2)	2 (2.0)	7 (12.7)	0 (0.0)
Increased ALP	14 (14.1)	0 (0.0)	6 (10.9)	2 (3.6)
Hypercholesterolemia	11 (11.1)	0 (0.0)	2 (3.6)	0 (0.0)
Hypertriglyceridemia	11 (11.1)	0 (0.0)	2 (3.6)	0 (0.0)
Anemia	7 (7.1)	0 (0.0)	4 (7.3)	1 (1.8)
Increase bilirubin	5 (5.1)	1 (1.0)	1 (1.8)	0 (0.0)
Hyperglycemia	1 (1.0)	0 (0.0)	3 (5.5)	1 (1.8)

*γ-GT* γ-glutamyltranspeptidase, *ALT* alanine transaminase, *AST* aspartate transaminase, *ALP* alkaline phosphatase

^a^Some patients had experienced several AEs

Treatment-related grade 3–4 AEs occurred in 51 (51.5%) patients in the famitinib group and 20 (36.4%) in the placebo group (Table 4). The most frequent famitinib-related grade 3–4 AEs were hypertension, hand-foot syndrome, thrombocytopenia, neutropenia, proteinuria, and liver dysfunction. The most frequent AEs leading to dose modification were dermatological, gastrointestinal, and metabolic or laboratory events.

Serious adverse events (SAEs) occurred in 11 (11.1%) patients in the famitinib group and 5 (9.1%) in the placebo group (*P* = 0.788). The most notable SAE in the famitinib group was intestinal obstruction (*n* = 5). Other SAEs included infection, hemoptysis, hypertension, fatigue, renal failure, upper gastrointestinal hemorrhage, and hepatic encephalopathy in the famitinib group, whereas cerebral infarction, cerebral hemorrhage, fatigue, and thrombocytopenia were observed in the placebo group. The majority of SAEs were resolved, whereas four patients died of SAEs (three in the famitinib group and one in the placebo group).

## Discussion

VEGF and its receptors play a critical role in angiogenesis in CRCs [18–24]. Bevacizumab is a humanized monoclonal antibody designed to block VEGF and has shown efficacy on mCRC [25–27]. Sorafenib and sunitinib, two small-molecule TKIs, have been studied in the treatment of mCRC patients [28–32]. Regorafenib was approved to treat mCRC, with median OS prolongation by 1.4 months in the CORRECT study [10] and 2.5 months in the CONCUR study [11]. Recently, fruquintinib as third-line treatment was reported to prolong the survival of mCRC patients [12]. Based on the recent unpublished FRESCO study, the median OS was 9.3 months (95% CI, 8.2–10.5 months) in the fruquintinib group versus 6.7 months (95% CI 5.9–8.1 months) in the placebo group, in a total of 416 mCRC patients; the median PFS was 3.7 months (95% CI 3.6–4.6 months) in the fruquintinib group versus 1.8 months (95% CI 1.8–1.8 months) in the placebo group.

In the present study, the addition of famitinib to supportive care significantly prolonged PFS in patients with mCRC who had failed all standard chemotherapy agents with or without monoclonal antibody. The median PFS prolongation was 1.3 months by famitinib in the present study, 1.9 months by fruquintinib based on the unpublished FRESCO study, and 1.4 months by regorafenib in the CORRECT study [10], showing that VEGFR-blocking TKIs are effective in treating mCRC after chemotherapy failure. However, in the present study, famitinib failed to prolong the median OS, which may be related to that many patients in the placebo group received further anti-tumor therapy, especially targeted therapy. At the same time, we did observe a trend of survival prolongation in patients treated with famitinib who had been heavily pre-treated with systematic chemotherapy. The patients in the famitinib group with ≥ 6 cycles of first-line chemotherapy and ≥ 3 cycles of second-line chemotherapy had 2.0 months increase in median OS compared with patients in the placebo group (data not shown).

The median OS prolongation by famitinib in the present study was slightly shorter than those by regorafenib [10] and fruquintinib (data not published). The higher percentages of patients with ECOG score of 1 and elderly patients in the present study than those in the other two studies may influence OS. In addition, only 30.2% of patients had received anti-angiogenesis therapy before fruquintinib treatment in the unpublished FRESCO study, but 39.4% of patients had received anti-angiogenesis therapy before famitinib treatment in the present study, which might also influence OS.

The most frequent AEs related to famitinib were proteinuria, hand-foot syndrome, fatigue, and hypertension in the present study, which were consistent with those observed in a phase I study [14] and typical AEs of small-molecule VEGF TKIs [10, 11]. These AEs occurred frequently during the early course of treatment and were generally manageable with dose reduction or interruption. The occurrence of hypertension could reflect the anti-angiogenesis effect of VEGF or VEGFR TKIs. The rate of hypertension caused by famitinib in the present study was higher than that caused by regorafenib [10], suggesting that famitinib may have stronger anti-angiogenesis effect than regorafenib. Another multiple-target TKI sunitinib, with similar structure as famitinib, was reported that its efficacy on renal cell carcinoma was related to the occurrence of hypertension [33]. It is worth exploring if there is correlation between the efficacy and hypertension in famitinib treatment in further study.

One limitation of the present study is that the primary tumor site and *RAS/*v-Raf murine sarcoma viral oncogene homolog B1 (*BRAF*) mutation were not included in subgroup analysis because of the small sample size.

## Conclusions

In summary, famitinib significantly prolonged the median PFS for patients with refractory mCRC who had failed two or more lines of standard chemotherapy, and the toxicities were tolerable. A phase III trial is warranted in further study.

## Abbreviations

5-FU: 5-fluorouracil
AEs: adverse events
CR: complete response
DCR: disease control rate
ECOG: Eastern Cooperative Oncology Group
EORTC: European Organization for Research and Treatment of Cancer
FAS: full analysis set
FLT3: FMS-like tyrosine kinase-3 receptor
HR: hazard ratio
IQR: interquartile range
IRB: institutional review board
LDH: lactate dehydrogenase
mCRC: metastatic colorectal cancer
MMRM: mixed model for repeated measurements
mOS: median overall survival
ORR: objective tumor response rate
OS: overall survival
PD: disease progression
PFS: progression-free survival
QoL: quality of life
rTKI: receptor tyrosine kinase inhibitor
SAE: serious adverse events
VEGFR-2 and 3: vascular endothelial growth factor receptor-2 and 3
